# Advanced sleep disorder detection using multi-layered ensemble learning and advanced data balancing techniques

**DOI:** 10.3389/frai.2024.1506770

**Published:** 2025-01-28

**Authors:** Muhammad Mostafa Monowar, S. M. Nuruzzaman Nobel, Maharin Afroj, Md Abdul Hamid, Md Zia Uddin, Md Mohsin Kabir, M. F. Mridha

**Affiliations:** ^1^Faculty of Computing and Information Technology, King Abdulaziz University, Jeddah, Saudi Arabia; ^2^Department of Computer Science and Engineering, Bangladesh University of Business and Technology, Dhaka, Bangladesh; ^3^Sustainable Communication Technologies, SINTEF Digital, Oslo, Norway; ^4^Superior, Polytechnic School, University of Girona, Girona, Spain; ^5^Department of Computer Science and Engineering, American International University, Dhaka, Bangladesh

**Keywords:** machine learning, sleep disorder, ensemble approach, explainable AI, healthcare, diagnosis, ensemble models

## Abstract

Sleep disorder detection has greatly improved with the integration of machine learning, offering enhanced accuracy and effectiveness. However, the labor-intensive nature of diagnosis still presents challenges. To address these, we propose a novel coordination model aimed at improving detection accuracy and reliability through a multi-model ensemble approach. The proposed method employs a multi-layered ensemble model, starting with the careful selection of N models to capture essential features. Techniques such as thresholding, predictive scoring, and the conversion of Softmax labels into multidimensional feature vectors improve interpretability. Ensemble methods like voting and stacking are used to ensure collaborative decision-making across models. Both the original dataset and one modified using the Synthetic Minority Oversampling Technique (SMOTE) were evaluated to address data imbalance issues. The ensemble model demonstrated superior performance, achieving 96.88% accuracy on the SMOTE-implemented dataset and 95.75% accuracy on the original dataset. Moreover, an eight-fold cross-validation yielded an impressive 99.5% accuracy, indicating the reliability of the model in handling unbalanced data and ensuring precise detection of sleep disorders. Compared to individual models, the proposed ensemble method significantly outperformed traditional models. The combination of models not only enhanced accuracy but also improved the system's ability to handle unbalanced data, a common limitation in traditional methods. This study marks a significant advancement in sleep disorder detection through the integration of innovative ensemble techniques. The proposed approach, combining multiple models and advanced interpretability methods, promises improved patient outcomes and greater diagnostic accuracy, paving the way for future applications in medical diagnostics.

## 1 Introduction

Sleep is a physiological necessity that may revitalize and repair the body. Furthermore, obtaining high-quality sleep is essential for maintaining good health (Šušmáková, 2004; Thorpy, 2017). Numerous physical and emotional health issues can result from poor sleep (Walker, 2017). In traditional sleep evaluation, the patient is required to sleep in a testing room and sensors are connected to their bodies to measure biological signals, such as electroencephalogram (EEG), Electrooculography, Electromyography (EMG), etc. (Hafezi et al., 2020). Trained sleep technologists, whether experts or licensed, can accurately pinpoint sleep issues by analyzing physiological data collected from patients through Polysomnography (PSG) to find sleep stage intervals and irregularities that may be signs of sleep disorders. The American Academy of Sleep Medicine's sleep scoring guidelines were used in this process (Berry et al., 2012).

However, a range of sleep-related problems such as sleep apnea (Mostafa et al., 2019), insomnia (Shahin et al., 2017), and REM sleep behavior disorder (RBD) (Lee et al., 2022) can diminish sleep quality. A multitude of detrimental health issues, such as daytime sleepiness (Shernazarov, 2023), headaches (Jansen et al., 2019), and weakened immunity, can all be made more likely by sleep disturbances. As sleep disorders are rising, it is crucial to identify these issues through thorough monitoring of sleep patterns accurately (Bazilio et al., 2019).

The problem revolves around the crucial role of sleep in maintaining good health and the rising prevalence of sleep disorders like sleep apnea and insomnia. Conventional diagnostic procedures have significant limitations, including high costs and inconvenience due to the time-consuming process of attaching multiple sensors. Additionally, sleep technologists need to manually annotate and interpret PSG recordings, which encompass extensive data.

Electrocardiography (ECG) is a physiological signal that can reflect cardiac activity (Hafezi et al., 2020; Hilal et al., 2023). ECG is considered an alternative physiological source for healthcare technology because it has the most informative signal, including cardiac rhythm, breathing activity, and ECG-derived respiratory activity (Tripathi et al., 2022). ECG has been used in specific research to automatically identify sleep abnormalities, such as sleep apnea (Erdenebayar et al., 2019; Bernardini et al., 2021) and insomnia (Shahin et al., 2018). All these factors, including personal characteristics like gender, age, and employment status, as well as metrics such as sleep duration, subjective sleep quality assessments, daily physical activity levels, stress levels, BMI categories, blood pressure readings, resting heart rates, and daily step counts, can serve as predictors for sleep disorders (Ayanaw et al., 2022). LeCun et al. (2010) suggested different machine learning-based detection techniques for a single sleep problem from one or more input sources. Numerous machine learning and deep learning techniques, including Support Vector Machines (SVM), Artificial Neural Networks (ANN), and Convolutional Neural Networks (CNN), were employed in these investigations. This research also used manually created feature sets that were retrieved utilizing standard machine-learning techniques. Nonetheless, research that uses multiclass classification and can automatically classify sleep disorders ought to be mandated.

Traditional sleep disorder diagnostic procedures, such as PSG, are expensive and labor-intensive, limiting their scalability and usefulness. Machine learning and ensemble learning present a viable alternative, enabling for the study of large datasets to reveal complex patterns and predictors of sleep disorders. The addition of a new sleep disorder dataset improves diagnostic accuracy, while ensemble learning approaches increase resilience and dependability. By using these developments, we may transform sleep disorder detection, provide doctors with relevant information, and ultimately enhance patient outcomes. Our sleep disorder detection study is motivated by the severe health consequences associated with diseases such as sleep apnea, insomnia, and other disorders. These illnesses can cause various health consequences, including cardiovascular disease and cognitive impairment, affecting general well-being. Since, traditional diagnostic approaches, such as polysomnography testing, are costly and time-consuming, also have access restrictions; consequently, there is a need for non-invasive, cost-effective detection technologies. Our study builds upon these foundational works by introducing a novel coordination model that utilizes ensemble learning techniques to further enhance diagnostic reliability and effectiveness. The model demonstrates remarkable performance by adopting a multi-layered ensemble approach and innovative methodologies such as thresholding and predictive scoring. The method effectively addresses challenges associated with unbalanced data through techniques like SMOTE evaluation. These findings represent a significant advancement in the field, promising improved diagnostic capabilities and, ultimately, better patient outcomes.

The contribution of the study on Sleep disorders can be summarized as follows:

- The ensemble model consistently achieves high accuracy, establishing it as a reliable and powerful diagnostic tool for healthcare professionals.
- With softmax labels and comprehensive feature analysis, the model enhances understanding of sleep-related conditions, benefiting healthcare providers and patients.
- Demonstrating precision across various classes, including insomnia, sleep apnea, and regular sleep, the model showcases its versatility and applicability in diverse clinical scenarios.
- Setting a precedent for integrating machine learning into healthcare, the model demonstrates the potential of data-driven approaches to revolutionize diagnostics and improve patient outcomes.

## 2 Related works

Fifty to seventy million Americans suffer from sleep disorders such as sleep apnea, parasomnias, and hypersomnias (Hillman et al., 2006). For the diagnosis of sleep disorders, overnight polysomnography (PSG), which includes EEG brain monitoring, is crucial. PSG can be automated by the recent development of complex neural network learning algorithms and large physiological datasets, opening access to expert-level sleep analysis. SleepNet, a deployable annotation tool for sleep staging, is based on sleep EEG neural networks. SLEEPNET employs a deep recurrent neural network that was trained on PSGs from more than 10,000 patients at the MGH Sleep Laboratory, one of the largest datasets for sleep physiology. Similar to expert-expert IRA, SLEEPNET achieves human-level annotation on an independent test set of 1,000 EEGs with 85.76% accuracy and 79.46% algorithm-expert inter-rater agreement (IRA) (Biswal et al., 2017). Both research endeavors (Fraiwan and Lweesy, 2017; Koolen et al., 2017) seek to streamline the analysis of neonatal sleep states using EEG recordings. The initial study demonstrates an overall accuracy of 80.4%, while the second study surpasses this with an accuracy of 85%. Moreover, it achieves a sensitivity of 83% and specificity of 87% in distinguishing between quiet and active sleep epochs. Radha et al. (2021) trained a deep recurrent neural network to classify sleep stages (wake, rapid-eye-movement, N1/N2, and N3) using electrocardiogram (ECG) data from 292 subjects and 584 recordings. The domain and decision combination transfer learning technique yielded the best results (Cohen's kappa of 0.65 ± 0.11, accuracy of 76.36 ± 7.57%), surpassing PPG and ECG baselines. The performance of this PPG-based 4-class sleep stage categorization surpasses any found in existing literature, marking a significant advancement and bringing home sleep stage monitoring closer to clinical application. Classifying sleep states serves as an initial step in screening for sleep disorders. However, manually performing this task is laborious and time-intensive for specialists. Many studies examined automated polysomnogram signal analysis. They found that support vector machines with radial basis function and random forest can predict sleep stages and feature-based neural networks with state-of-the-art performance (Sekkal et al., 2022).

The first deep learning method for sleep stage classification was introduced by Chambon et al. (2018). It uses all multivariate and multimodal polysomnography (PSG) inputs (EEG, EMG, and EOG) and learns end-to-end without spectrogram computing or manual feature extraction. The initial stage of each method is learning linear spatial filters to increase the signal-to-noise ratio using an array of sensors. On the other hand, the representation of a softmax classifier is given by the last layer. Several spatiotemporal distribution insights for signals of interest derive from their investigation: they use six EEGs with two EOG (left and right) and three EMG chin channels for the best classification performance with balanced accuracy.

Rempe et al. (2015) presented a semiautomated method for evaluating rodent sleep disorder using EEG and EMG signals. Manual scoring by eye inspection is time-consuming and uses arbitrarily segmented epochs. Using principal component analysis and naïve Bayes classification with EEG and EMG inputs, this system was verified using human-scored data from C57BL/6J and BALB/CJ mice. The machine scoring method correctly detected wake and slow-wave sleep (SWS) states in over 89% of epochs. The algorithm correctly detected most rapid-eye-movement sleep (REMS) epochs, but some were misclassified as SWS or wake. Koch et al. (2014) proposed a new data-driven technique that uses spectral EEG, EOG, and ocular correlation in 1-s windows. The model is evaluated on controls, PLM, iRBD, and Parkinson's patients. Optimized with 50 participants and validated on 76 patients, the model has 68.3% subject-specific accuracy and 67.2%–70.1% group-specific accuracy. This computer is capable of analyzing EEGs in real time. For offline analysis, specific samples are stored on disk following continuous visual, analog, and tabular data analysis. Pattern recognition predicts sleep-awake phases using wave frequency distribution. An independent channel can confirm results. Averaging and clustering disk samples permit statistical EEG signal comparisons. Using dexmedetomidine as a prototype drug, it predicted deep hypnotic levels with 81% accuracy and 0.89 AUC (Nagaraj et al., 2020). The strategy significantly increases sleep stage classification accuracy and explains multi-class labeling of univariate EEG signals by identifying key signal components. They tested the approach on the sleep-EDF dataset and achieved 86.8% accuracy. With the fewest examples, essential sleep stage N1 classification accuracy was 16.3% greater than state-of-the-art machine learning (Dutt et al., 2023).

TinySleepNet (Supratak and Guo, 2020) introduced an efficient deep-learning network and a novel end-to-end training strategy for automatic sleep stage grading using raw single-channel EEG data. Due to fewer model parameters, their model requires less training data and processing. Their training strategy influences data augmentation to shield the model from time axis shifts and prevent sleep stage memory. Seven public sleep datasets with different scoring criteria, recording channels, and settings were tested. Yan et al. (2021) proposed an end-to-end deep learning architecture using raw polysomnographic recordings to automate sleep assessment. The model uses 2D-CNNs to automatically learn features from multi-modality inputs and a “squeeze and excitation” block to recalibrate channel-wise feature responses. A softmax classifier makes Final sleep stage predictions using the learned representations. SHHS and Sleep-EDF public sleep datasets with different channels are used to evaluate the model. Their findings revealed that their model achieved an accuracy of 85.2% on the SHHS dataset and 85% accuracy on the Sleep-EDF dataset. The deep learning model with convolutional neural networks and long short-term memory units performed well. Werth et al. (2020) assessed three datasets of 34 preterm children and 18,018 meticulously annotated 30-s sleep episodes. These annotations included active, quiet, intermediate, awake, and caretaking sleep states. The study explored four recurrent neural network architectures for two, three, and all-state analyses. Specifically, a sequential network was compared with gated recurrent unit and long- and short-term memory models. ResNet, ResNext, and other architectures also utilized residual connections to enhance depth. Notably, the essential sleep active and quiet, demonstrated a kappa value of 0.43 ± 0.08. Goshtasbi et al. (2022) proposed SleepFCN, which uses multi-scale feature extraction (MSFE) and residual dilated causal convolutions (ResDC) for feature extraction and temporal sequence encoding. After this, one-sized kernel convolutional layers replace dense layers to construct the fully convolutional neural network. Since sleep stages are unevenly distributed, they weight our loss function by the number of samples in each class. SleepFCN was tested using the Sleep-EDF and SHHS datasets.

Using dexmedetomidine as a prototype drug, it predicted deep hypnotic levels with 81% accuracy and 0.89 AUC (Nagaraj et al., 2020). The strategy significantly increases sleep stage classification accuracy and explains multi-class labeling of univariate EEG signals by identifying key signal components. They tested the approach on the sleep-EDF dataset and achieved 86.8% accuracy in detecting five sleep stages. With the fewest examples, essential sleep stage N1 classification accuracy was 16.3% greater than state-of-the-art machine learning (Dutt et al., 2023). In a different dataset, Chen et al. (2022) investigated combining *z*-scoring with deep learning. Using 12 three-hour EEG/EMG recordings from sleeping mice, the open-source program Accusleep identified sleep states using a combination of *z*-scoring and deep learning via a convolutional neural network. Cohen's *k* with an accuracy range of 0.66–0.71% and 85–92%.

The strategy significantly increases sleep stage classification accuracy and explains multi-class labeling of univariate EEG signals by identifying key signal components. They tested the approach on the sleep-EDF dataset and achieved 86.8% accuracy in detecting five sleep stages. With the fewest examples, essential sleep stage N1 classification accuracy was 16.3% greater than state-of-the-art machine learning (Dutt et al., 2023). In Sathyanarayana et al. (2016), multilayer perceptrons (MLP), convolutional neural networks (CNN), simple Elman-type recurrent neural networks (RNNs), long short-term memory (LSTM-RNN), and a time-batched variant of LSTM-RNN (TB-LSTM) are compared with conventional logistic regression. According to the results, deep learning models performed better than conventional logistic regression. Notably, CNN outperforms logistic regression overall under the ROC curve by 0.9449—46% better than the prior. It also showed the highest specificity and sensitivity.

Loh et al. (2020) discussed the importance of sleep for well-being and the rise of sleep disorders worldwide. Sleep analysis is essential for recognizing sleep problems, but skilled visual interpretation creates variability. A Programmed Diagnostic Tool (PDT) based on artificial intelligence, notably deep learning (DL), for timely sleep disturbance diagnosis is proposed to address this.

Masood et al. (2018) developed a system that trains deep learning models using MDT measurements. This setup enables prompt detection and isolation of network anomalies or cell outages, thereby reducing the self-healing duty cycle of Self-Organizing Network (SON). After reviewing the recent methods outlined in Table 1, illustrating various approaches within this domain alongside the corresponding research gaps or challenges encountered during their implementation, we found inspiration to address these gaps and bridge the existing divides. Concerns were raised regarding studies conducted in controlled environments, imbalanced datasets, and movement artifacts, indicating opportunities for improvement in real-world sleep pattern understanding and algorithm robustness. These gaps motivated our work to address these challenges and advance sleep analysis methodologies.

**Table 1:** Comprehensive comparative analysis: strengths and weaknesses of current approaches in several domains.

**References**	**Focused methods**	**Signals used**	**Challenges/ research gap**
Zhang et al. (2021)	Deep CNN-LSTM	ECG signals	Has limitations, including challenges in detecting hypopnea events, scoring transition epochs, and plans for future enhancements in discriminating arousal and non-event epochs
Shahin et al. (2017)	DNN, DNN-HMM	EEG	Lacked scope of other sleep disorders
Jarchi et al. (2020)	SVM, LSVM, Autocarousel, Random Forest, KNN, XGB, and multilayer perceptron	ECG and EMG	Absent a greater variety of biosignals
Hafezi et al. (2020)	CNN + LSTM + Fully connected Layer	Tracheal movements	The investigation is carried out in a controlled environment.
Hafezi et al. (2019)	Portable system based on accelerometer, CNN, RNN + LSTM, CNN + LSTM, and recording tracheal motions	NA	Imbalanced dataset and movement artifacts

## 3 Methodology

**Figure 1:**
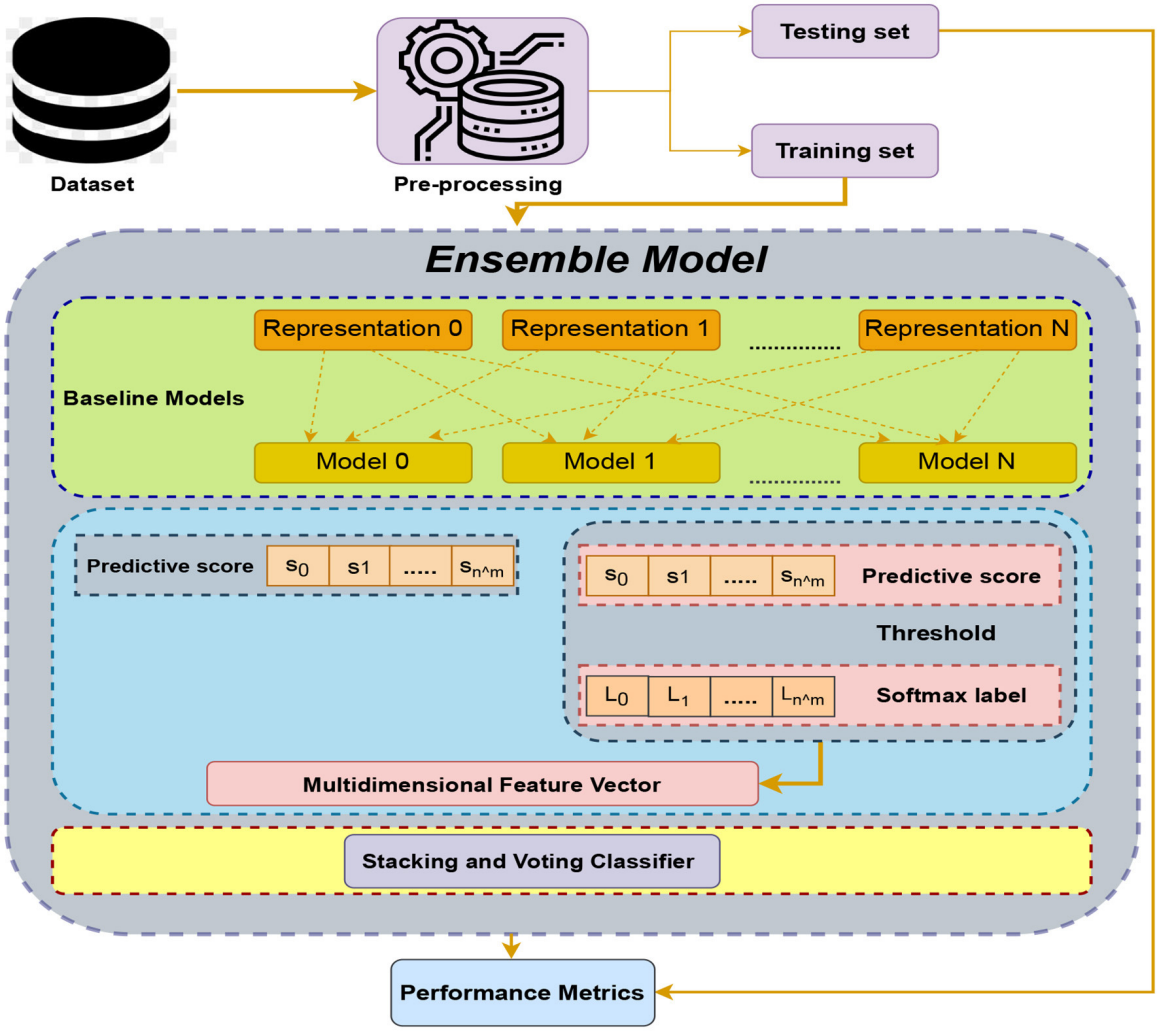
Comprehensive workflow illustrating the architecture for sleep disorder detection.

Ensemble learning has been performed through the use of random forest, SVM, logistic regression, KNN, XGBoost, and voting classifier. Representation is a crucial aspect of this process. This suggests that the ensemble learning technique involves combining the predictions of multiple machine learning models, including random forest, SVM, logistic regression, KNN, XGBoost, and a voting classifier. Figure 1 depicts the intricate structure of the proposed model utilized in our sleep disorder detection technique.

### 3.1 Dataset description

Broader factors related to sleep and daily routines were covered by 13 columns that comprise the Sleep Health and Lifestyle dataset. Physical activity, stress levels, age, sex, occupation, blood pressure, heart rate, daily steps, sleep duration, quality, and presence or absence of sleep disorders were all included in the dataset. Comprehensive investigations of cardiovascular health, lifestyle factors, sleep disorders and sleep metrics are made possible by key features of the dataset. Many details are provided by dataset columns, including BMI category, blood pressure readings, resting heart rate, daily step count, sex, age, occupation, sleep length, subjective sleep quality ratings, and presence or absence of sleep disorders. The sleep disorder column, for example, lists “None” for those without a specific sleep disorder, “Insomnia” for those with trouble falling or staying asleep, and “Sleep apnea” for those with breathing problems. dangerous to their health.

### 3.2 Dataset analysis and discussion

**Figure 2:**
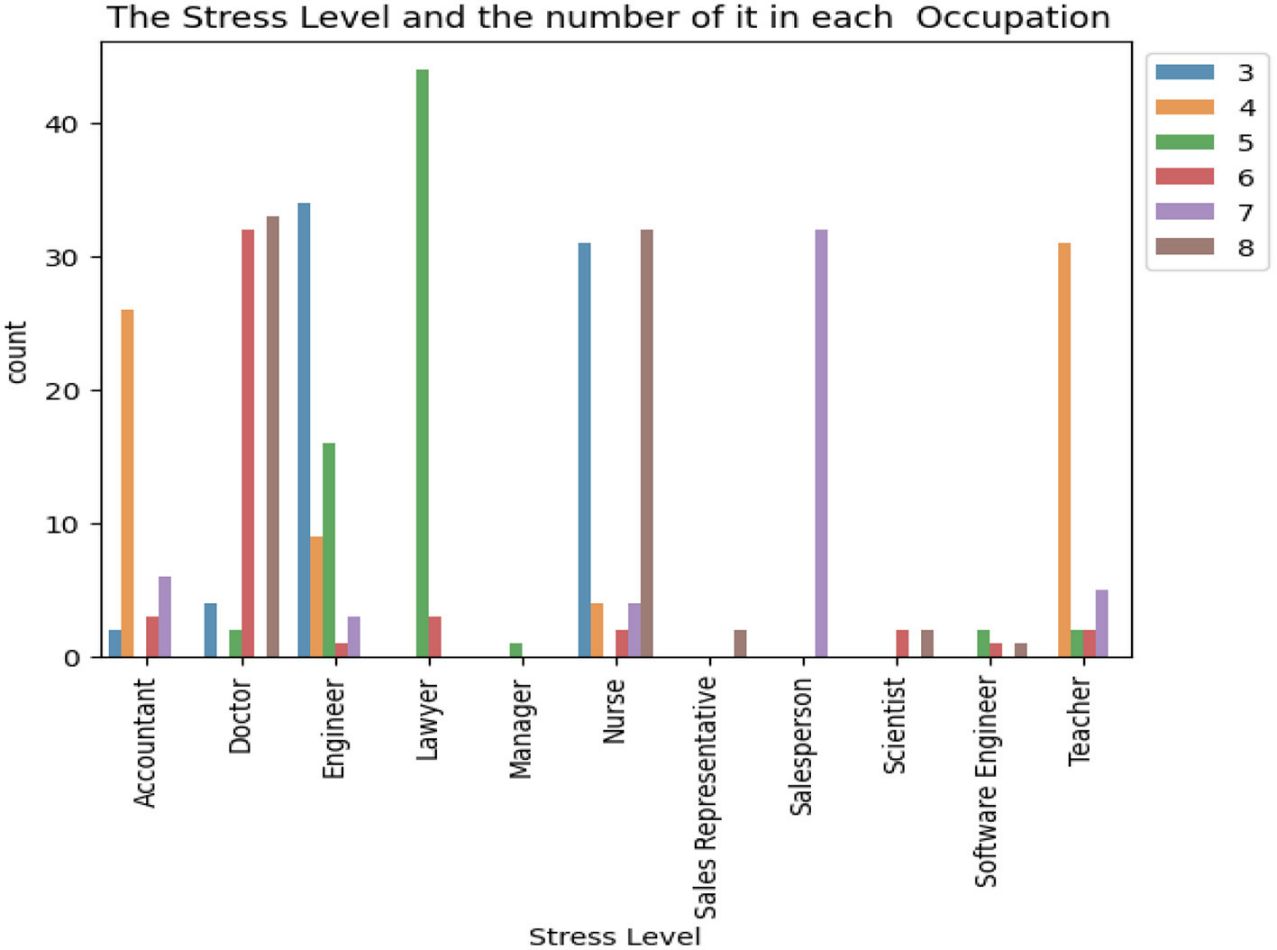
A comprehensive view of the distribution of sleep disorders based on occupation.

Figure 2 illustrates the distribution of the stress levels categorized by occupation. The dataset encompasses 11 distinct occupational categories, which include accountants, doctors, engineers, lawyers, managers, nurses, sales representatives, salespersons, scientists, software engineers, and teachers. Notably, the analysis reveals that lawyers exhibit the highest incidence of stress level 5, whereas sales representatives report the lowest occurrence of stress. Also, Doctors and Engineers have almost every aspects of stress level.

**Figure 3:**
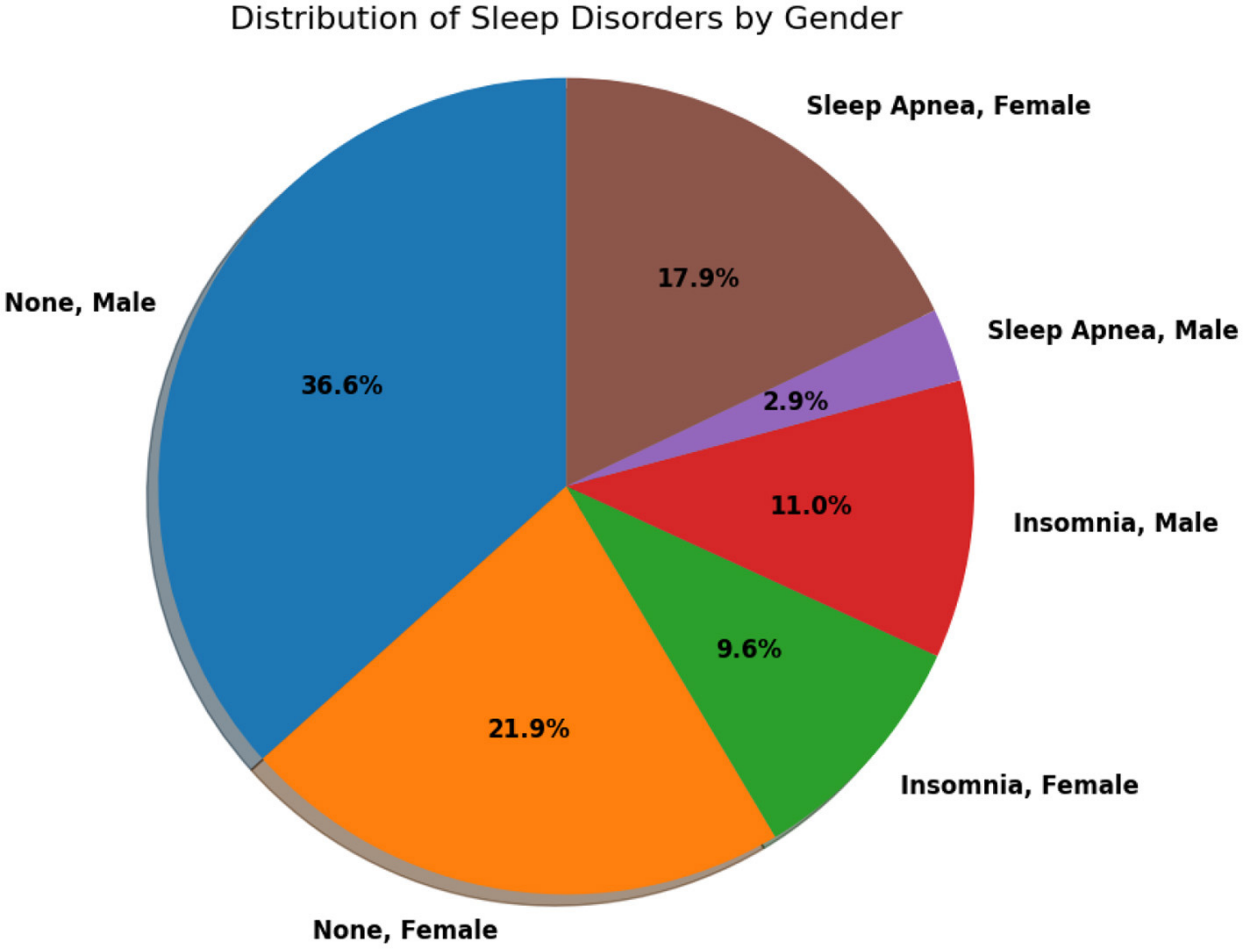
A comprehensive view of the distribution of sleep disorders based on gender.

Figure 3 provides a comprehensive view of the distribution of sleep disorders based on sex. This visual representation offers valuable insights into the prevalence and patterns of sleep-related issues within different sex groups. Analyzing the distribution illustrated in the figure can significantly enhance our understanding of the nuanced aspects of sleep disorders, facilitating the development of tailored interventions or strategies.

### 3.3 Data preprocessing processes

In the preprocessing phase, a collection of sophisticated methodologies was utilized to improve the caliber of the dataset. To preserve the original distribution while adjusting numbers to fit inside a certain range, we utilized the Min-Max Scaler. This was especially advantageous considering the varied magnitudes and existence of anomalies in our dataset. Equation 1 is the Min-max formula, where m represents the new value, x represents the original cell value, xmin represents the minimum value of the column, and xmax represents the maximum value of the column.


(1) $$\begin{array}{l} m=\frac{\left(x-xmin\right)}{\left(xmax-xmin\right)} \end{array}$$

To address the issue of missing values, particularly in time series data, we used many techniques, such as mean imputation and standardization. These measures not only addressed the missing data in our dataset but also established a stronger and more consistent basis for future analysis. Standardization is the process of transforming the signal of each data channel into a random variable with a mean of 0 and a variance of 1. This is achieved by using the sample mean (*m*) and sample variance (*s*), as shown in Equation 7.

**Figure 4:**
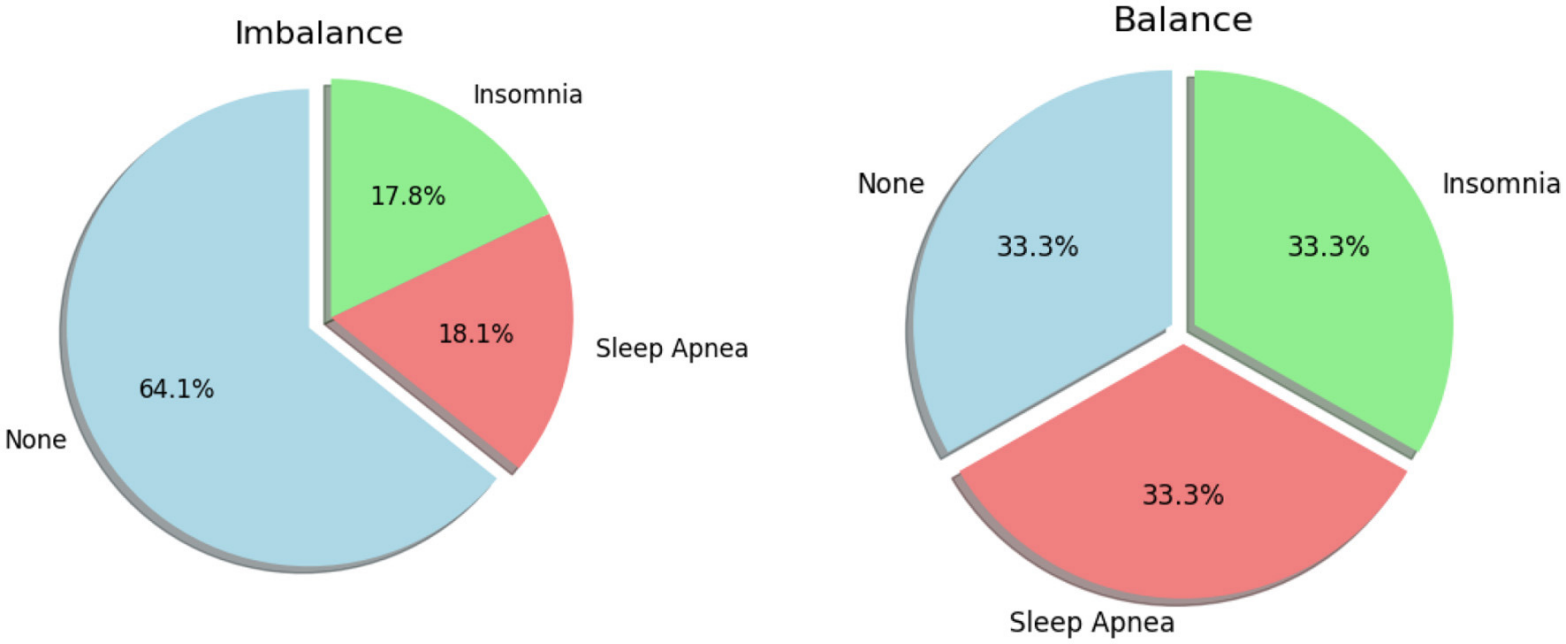
Graphical representation of balanced and imbalanced data distribution.

**Figure 5:**
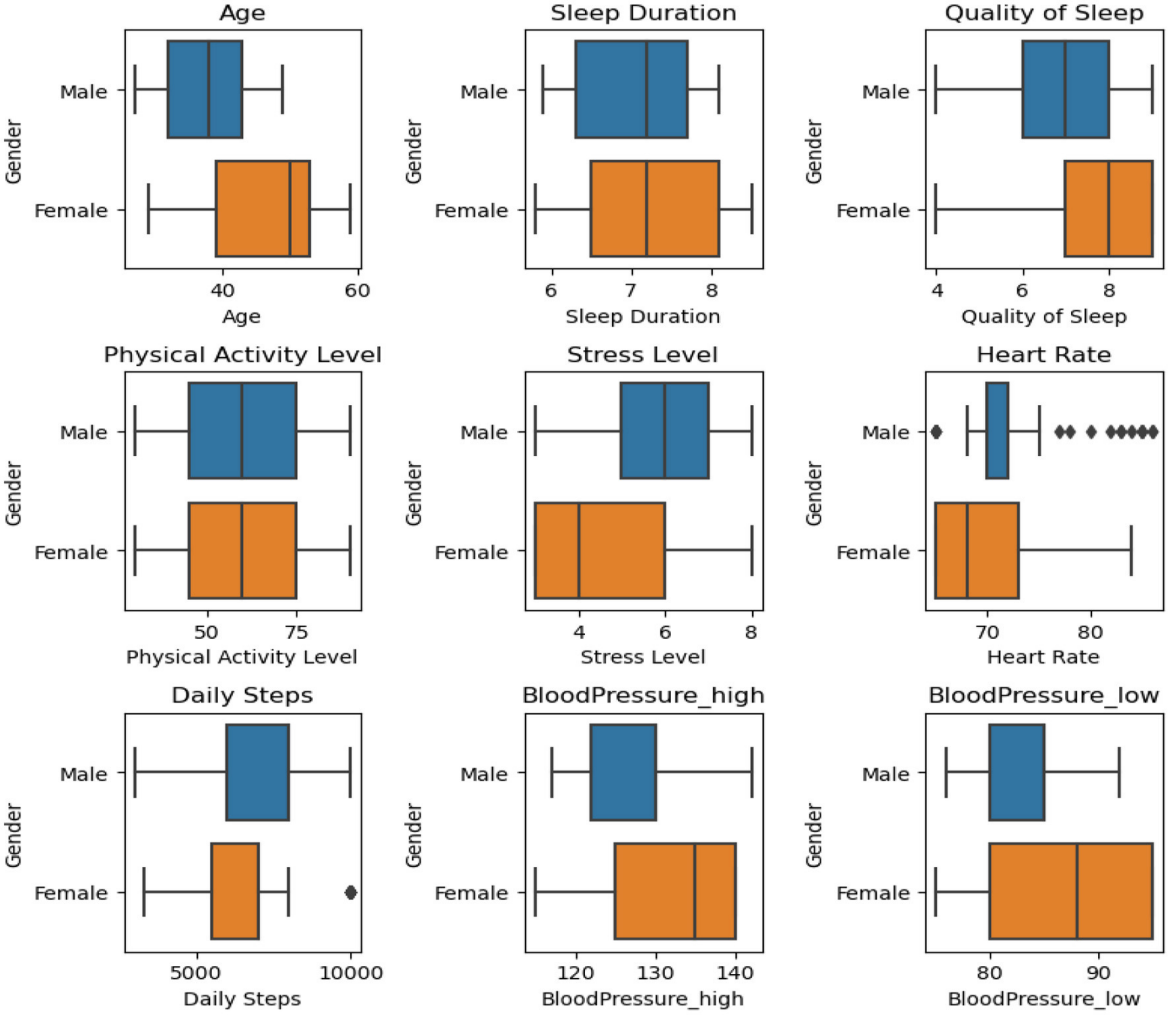
Boxplot analysis showing differences between genders across different trait data structures.

After undergoing preprocessing efforts, the dataset was refined, balanced, and ready to be seamlessly integrated with machine learning models. Figure 4 shows a graphical depiction of the balanced data. The dataset exhibited a pronounced imbalance, with 17.8% representing Insomnia, 18.1% for Apnea, and a substantial majority of 64.1% corresponding to “None” class. This imbalance posed a risk of model bias toward the majority class, potentially compromising performance on the minority classes. Subsequently, the Synthetic Minority Over-sampling Technique (SMOTE) was applied, successfully achieving a balanced distribution across all three classes, each accounting for 33.3% of the dataset. The introduction of synthetic samples through SMOTE effectively mitigated the initial imbalance, providing the model with a more equitable representation of each class. This balanced dataset is anticipated to enhance the model's training, reducing the risk of bias toward any specific class and improving its generalization capabilities. The equal distribution among classes ensures that the model can make predictions across all categories with increased accuracy and fairness. These thorough measures emphasize our dedication to guaranteeing the excellence and dependability of the data supporting our assessments. We carefully partitioned our data to provide a rigorous assessment of our model. For the purpose of training, we assigned 70% of the dataset. The validation set, which accounted for 20% of the data. The testing set, which made up 10% of the dataset. A series of critical steps has been implemented to ensure the effectiveness of our analysis. Initially, Figure 5 were utilized to visualize and identify potential outliers within our dataset, as outliers can significantly influence the accuracy of our predictive models. Following this, feature engineering techniques were applied to extract meaningful information from our data and enhance the predictive power of our models. Subsequently, outliers were removed from the dataset and the dataset was increased to mitigate their potential impact on model performance. By incorporating these steps into our analysis pipeline, accurate and reliable models are aimed at developing for the detection and diagnosis of sleep disorders.

The suggested approach for detecting sleep disorders utilizes an advanced voting classifier that combines four distinct machine learning algorithms: random forest, support vector machine (SVM), K-nearest neighbors (KNN), and XGBoost. This ensemble strategy is designed to enhance predictive performance by combining the strengths of multiple models.

The process begins with the training phase, where each base classifier is trained individually on the same training dataset. During this step, the Random Forest model learns to make predictions based on decision trees, SVM builds a hyperplane for classification, KNN categorizes samples based on the majority class of its nearest peers, and XGBoost, an optimized version of gradient boosting, focuses on reducing errors by learning from previous iterations.

Once the models are trained, the testing phase is initiated, where each classifier predicts the class of new, unseen test samples. For each test instance, class probabilities are generated by the models. These probabilities represent the likelihood of the sample belonging to each possible class (e.g., sleep disorder or non-disorder). In the voting mechanism, the ensemble combines the predictions of all four models. The class probabilities from Random Forest, SVM, KNN, and XGBoost are averaged using an equal weight voting strategy. This means that for each test sample, the final prediction is calculated by taking the mean of the probabilities from each classifier. The class with the highest aggregated probability is selected as the final predicted label.

By integrating different models through this majority voting system, the ensemble reduces the risk of overfitting that may arise from using a single model while also taking advantage of the complementary strengths of each algorithm. For example, Random Forest is known for robustness against overfitting, SVM performs well with clear margins between classes, KNN is effective in handling noise, and XGBoost excels in handling complex datasets with non-linear relationships. This ensemble model thus provides a more reliable and accurate prediction for sleep disorder detection with the detailed procedure outlined in Algorithm 1.

**Algorithm 1:**
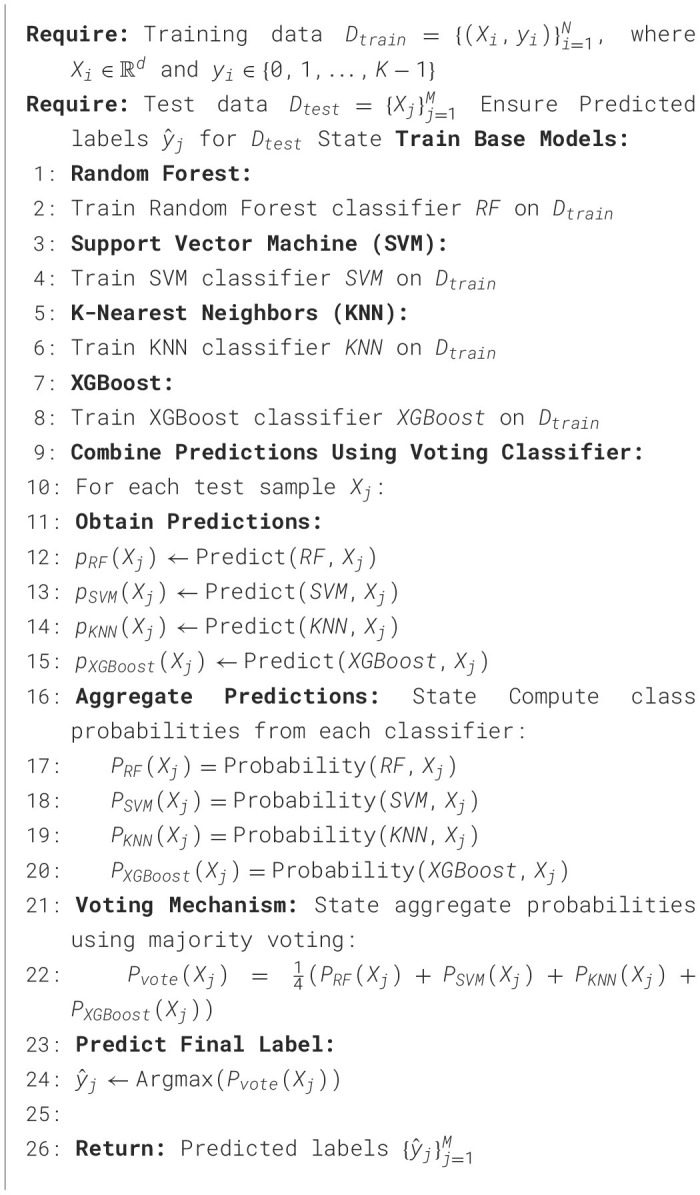
Advanced voting classifier combining random forest, SVM, KNN, and XGBoost.

### 3.4 Proposed model

**Figure 6:**
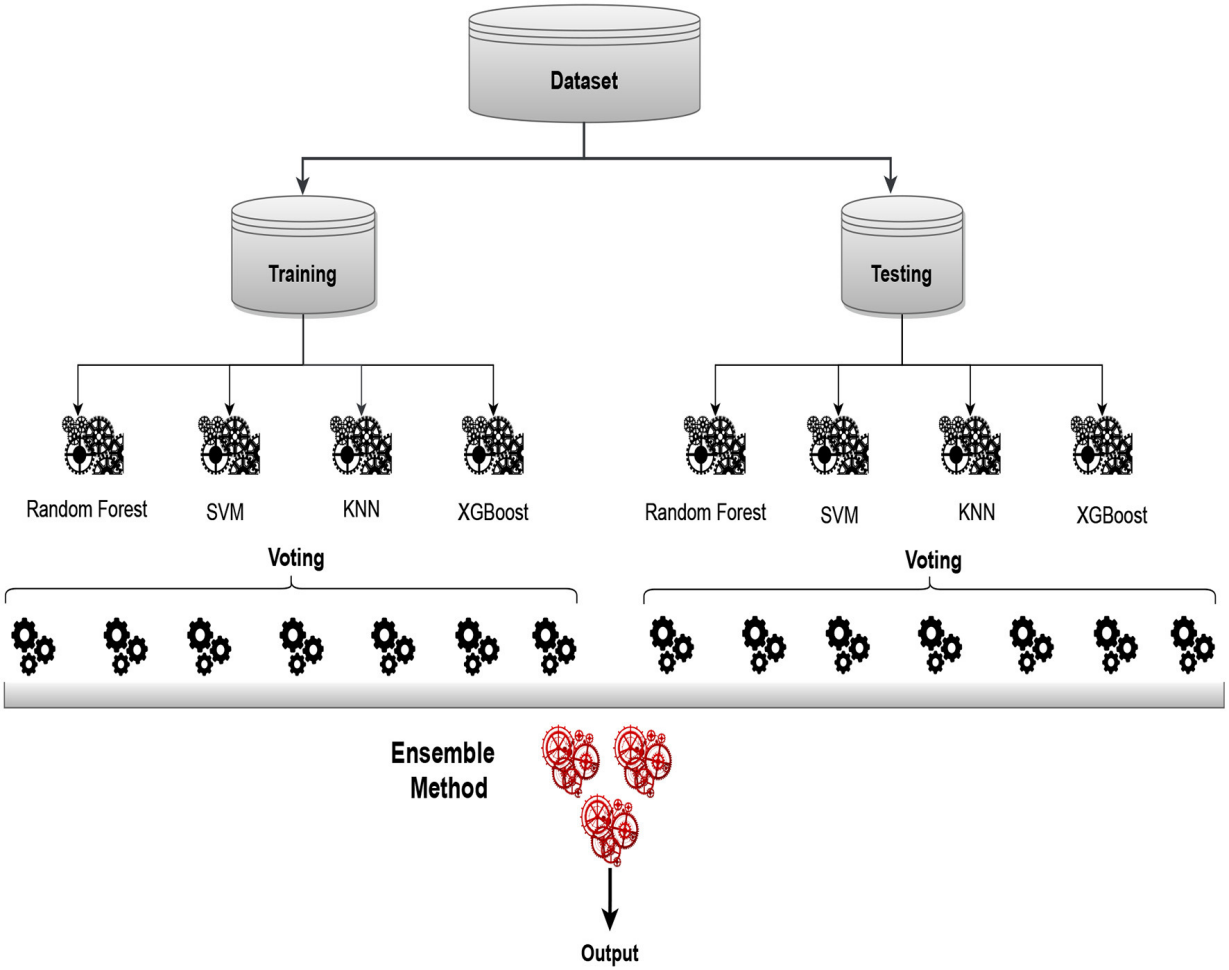
Proposed architecture of ensemble model utilizing voting classifier for sleep disorder detection.

Figure 6 of our approach involves selecting N models to form the baseline model representation. Subsequently, we follow a multi-layered process for predictive scoring and threshold determination. To enhance the interpretability of the results, we employ softmax labels, converting them into multidimensional feature vectors. A more thorough comprehension of the underlying patterns and relationships in the data is made possible by this transformation. In the final stages, we leverage ensemble techniques, specifically voting and stacking. Among these, the voting classifier emerges as the optimal choice, effectively combining the strengths of individual models. This strategic ensemble approach ensures a robust and accurate predictive framework, demonstrating superior performance compared to individual models. Through this comprehensive workflow, our methodology not only refines the predictive capabilities of the baseline models but also underscores the importance of ensemble strategies in achieving enhanced model performance.

### 3.5 Our ensemble approach

Our work used a sophisticated ensemble of machine learning techniques, including Random Forest, Support Vector Machine (SVM), k-Nearest Neighbors (kNN), and XGBoost. The ensemble is organized using a voting approach, with each model contributing to the final decision.


(2) $${\hat{y}}_{\text{ensemble}}=\text{MajorityVote}({\hat{y}}_{\text{RF}},{\hat{y}}_{\text{SVM}},{\hat{y}}_{\text{kNN}},{\hat{y}}_{\text{XGBoost}})$$

#### 3.5.1 Random forest

The random forest technique exploits the power of many decision trees to produce predictions that are both reliable and precise. To select random subsets of the training data using bootstrap sampling, a set of decision trees is constructed during the training phase. To ensure diversity, each tree is constructed using a distinct subset of features at each node. In the prediction phase, each tree “votes” for a particular class; the category that receives the most votes is selected as the winner. Random Forest has a reputation for handling a wide range of features and delivering top-notch results on multiple datasets. It combines multiple decision trees, and the final prediction probability for class *y* is determined by averaging the probabilities from individual trees:


(3) $$\begin{array}{l} P_{RF}\left(y\right)=\frac{1}{T}\overset{T}{\underset{i=1}{\sum }}P_{i}\left(y\right) \end{array}$$

where *T* is the number of trees in the Random Forest.

#### 3.5.2 Support vector machine

The state-of-the-art classification technique Support Vector Machine (SVM) finds the best hyperplane in the feature space to partition the classes. SVM finds the support vectors—the data points that are closest to the hyperplane—and determines the hyperplane that maximizes the margin between classes during the training phase. By mapping data into high-dimensional space, the kernel trick enables SVM to handle both linear and non-linear segmentation. SVM classifies new data points according to their position on the hyperplane during prediction. SVM works particularly well in high-dimensional spaces in situations where distinct class boundaries are required. Support Vector Machines (SVM) classify data points by finding the hyperplane that maximizes the margin between classes. The decision function for SVM is:


(4) $$\begin{array}{l} f_{SVM}\left(x\right)=\text{sign}\left(\overset{n}{\underset{i=1}{\sum }}\alpha_{i}y_{i}K\left(x,x_{i}\right)+b\right) \end{array}$$

where α_*i*_ are the coefficients, *y*_*i*_ are the class labels, *K* is the kernel function, and *b* is the bias term.

#### 3.5.3 K-nearest neighbors

A straightforward and intuitive method for classification is k-nearest neighbors, or KNN. All training examples are stored in memory by KNN during training. When a new data point is met during the prediction phase, KNN determines the distance between the new point and each training example. Based on these distances, it then chooses the k nearest neighbors and classifies the new location by majority vote among its neighbors. The simplicity and effectiveness of kNN in detecting local patterns in data is well recognized. Although it may be susceptible to misinformation or additional features. k-Nearest Neighbors (kNN) classifies data points based on the majority class among their k nearest neighbors. The prediction probability for class y is given by:


(5) $$\begin{array}{l} P_{kNN}\left(y\right)=\frac{1}{k}\overset{k}{\underset{i=1}{\sum }}\delta \left(y-y_{i}\right) \end{array}$$

where *k* is the number of neighbors, and δ is the Dirac delta function.

#### 3.5.4 XGBoost classifier

XGBoost is a popular and sophisticated algorithm known for its predictive modeling capabilities. Gradient boosting is thus used to build sequential decision trees, each tree correcting the mistakes from the previous one. XGBoost uses gradient boosting to reduce residual error during training by changing the weights of misidentified examples. The sum of the predictions from each tree gives the final prediction during the prediction phase. Because XGBoost is so good at identifying complex links in data, it is often used in both real-world and machine learning competitions. An ensemble learning technique called Extreme Gradient Boosting (XGBoost) combines the predictions of different decision trees. The prediction probability for class y is obtained by summing the contributions from all trees:


(6) $$\begin{array}{l} P_{XGB}\left(y\right)=\overset{n_{\text{trees}}}{\underset{i=1}{\sum }}f_{i}\left(x\right) \end{array}$$

where *n*_*trees*_ is the number of trees in the XGBoost model, and *f*_*i*_(*x*) represents the output of the *i*-th tree.


(7) $$\begin{array}{l} \tilde{x}=\frac{\left(x-\mu \right)}{\sigma } \end{array}$$

The Table 2 provides a comprehensive comparison of various machine learning models alongside their respective hyperparameters. Each row delineates a distinct model, including Random Forest, SVM, KNN, and XGBoost, while the corresponding columns detail the specific hyperparameters utilized in their configuration. Notable details include the number of estimators, criterion for splitting, maximum depth of trees, and other parameters crucial for model optimization. For instance, Random Forest employs parameters such as the number of estimators and the maximum depth, while SVM relies on parameters like the regularization parameter and kernel type. Similarly, KNN incorporates parameters like the number of neighbors and algorithm type, whereas XGBoost utilizes parameters such as the number of boosting rounds and maximum depth of trees. This table serves as a valuable resource for understanding the intricate configurations of each model, facilitating informed decision-making in the selection and fine-tuning of machine learning algorithms for various tasks.

**Table 2:** An analysis of hyperparameters and how they help to maximize model performance.

**Model**	**Hyper-parameter**
Random forest	*n*_estimators = 321, random_state = 42, criterion = “gini”, max_depth = 7, min_samples_split = 0.9, min_samples_leaf = 0.9, bootstrap = True, *n*_jobs = –1
SVM	*C* = 1.0, kernel = “poly”, degree = 5, gamma = “scale”
KNN	*n*_neighbors = 5, weights= “uniform,” algorithm = ”kd-tree”, *n*_jobs= –1
XGBoost	*n*_estimators = 249, random_state = 65, objective = “multi:softmax,” max_depth = 7, min_samples_split = 0.7, min_samples_leaf = 0.9, *n*_jobs = –1

## 4 Results and experiments

### 4.1 Result analysis

**Figure 7:**
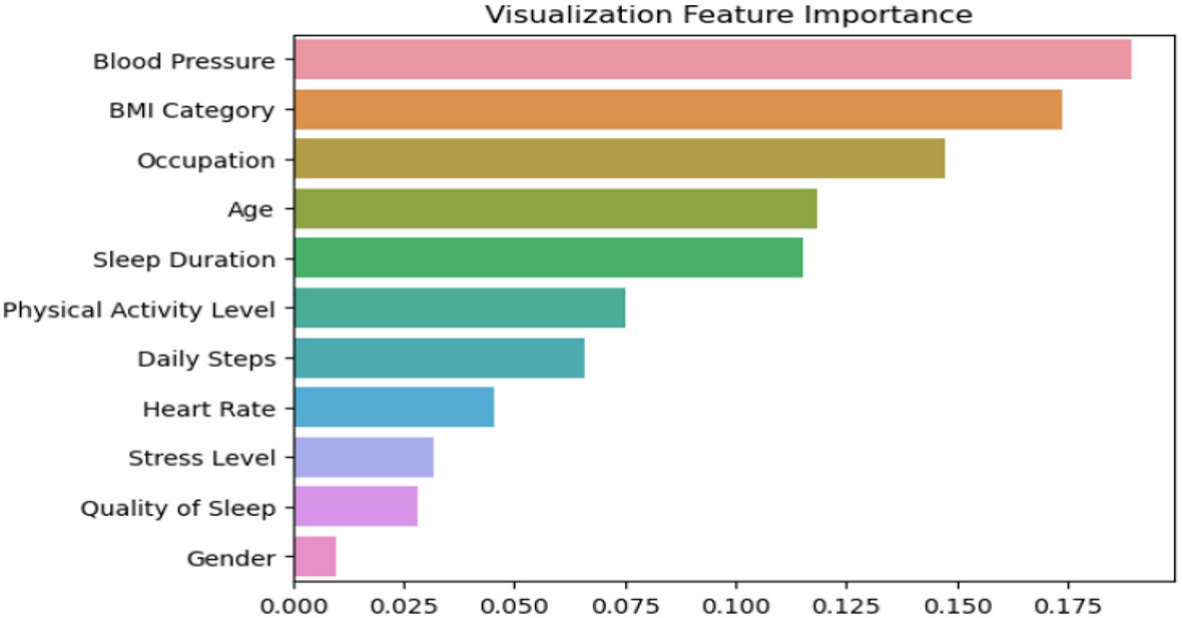
Feature importance analysis for our ensemble model, showing the contribution of each feature to the model's prediction performance.

Figure 7 depcits the feature importance analysis that provides valuable insights into the factors influencing sleep disorders, as observed in our proposed model aimed at enhancing accuracy. Notably, blood pressure emerges as the most crucial determinant, closely followed by BMI. Surprisingly, occupation secures the third position, as depicted in the figure. This thorough analysis of the 11 dataset features, encompassing variables such as age, gender, and heart rate, underscores gender as the least influential factor among them. These findings underscore the pivotal roles of blood pressure and BMI in our model's predictive capability for addressing sleep disorders.

#### 4.1.1 Performance metrics

Various standard evaluation metrics, such as accuracy, precision, recall, and F1-score, were employed to assess the system's performance. Accuracy, defined as the ratio of correctly classified samples to the total number of samples in the dataset, was utilized in the evaluation process:


(8) $$\begin{array}{l} Accuracy=\frac{TP+TN}{TP+FP+TN+FN} \end{array}$$

In summary, the number of true positive samples is represented by TP, true negative samples are denoted as TN, false positive samples are identified as FP, and false negative samples are quantified as FN.

Precision is the ratio of true positive samples to the total number of positive samples predicted by the model:


(9) $$\begin{array}{l} Precision=\frac{TP}{TP+FP} \end{array}$$

Recall, was calculated as the ratio of true positive samples to the total number of positive samples present in the dataset:


(10) $$\begin{array}{l} Recall=\frac{TP}{TP+FN} \end{array}$$

The F1-score is the harmonic mean of precision and recall and provides a balanced measure of the model's performance:


(11) $$\begin{array}{l} F1-score=2\frac{Precision*Recall}{Precision+Recall} \end{array}$$

Table 3 presents the performance metrics for various machine learning models trained on a standard dataset. The CatBoost model achieved a training accuracy of 93.20%, test accuracy of 91.13%, average precision of 0.93, average recall of 0.90, and an average F1-score of 0.91. Similarly, other models such as Gradient Boost, SVM, XGBoost, Logistic Regression, KNN, and Ensemble models were evaluated based on their accuracy, precision, recall, and average F1-score metrics.

**Table 3:** Performance metrics for various models before smote.

**Model**	**Train Acc**	**Test Acc**	**Average precision**	**Average recall**	**Average F1-score**
CatBoost	93.20	91.13	0.93	0.90	0.91
Gradient boost	93.36	90.34	0.95	0.97	0.92
SVM	88.80	87.96	0.93	0.89	0.90
XGBoost	94.28	91.13	0.96	0.91	0.93
Logistic regression	90.81	89.95	0.92	0.90	0.90
KNN	93.54	92.40	0.95	0.92	0.93
Ensemble model	95.75	94.96	0.96	0.96	0.96

Table 4 displays the results of applying the Synthetic Minority Over-sampling Technique (SMOTE) to the dataset. In this table, the Cat Boost model achieves a training accuracy of 94.88%, test accuracy of 93.18%, average precision of 0.94, average recall of 0.93, and an average F1-score of 0.93. Comparatively, it shows improvements in various metrics for most models, indicating that SMOTE positively impacts the model performance. Notably, our Ensemble model demonstrates a higher average F1-score of 0.97 in the SMOTE-applied dataset than 0.96 in the standard dataset. This suggests that SMOTE contributed to better generalization and overall performance in handling imbalanced datasets.

**Table 4:** Performance metrics for various models after smote.

**Model**	**Train Acc**	**Test Acc**	**Average precision**	**Average recall**	**Average F1-score**
Cat boost	94.88	93.18	0.94	0.93	0.93
Gradient boost	94.86	90.15	0.96	0.94,	0.93
SVM	89.33	87.88	0.91	0.92	0.91
XGBOST	94.86	92.42	0.95	0.93	0.92
Logistic regression	89.33	88.64	0.92	0.91	0.90
KNN	94.92	93.76	0.94	0.91	0.92
Our Ensembled	96.88	95.95	0.98	0.97	0.97

**Figure 8:**
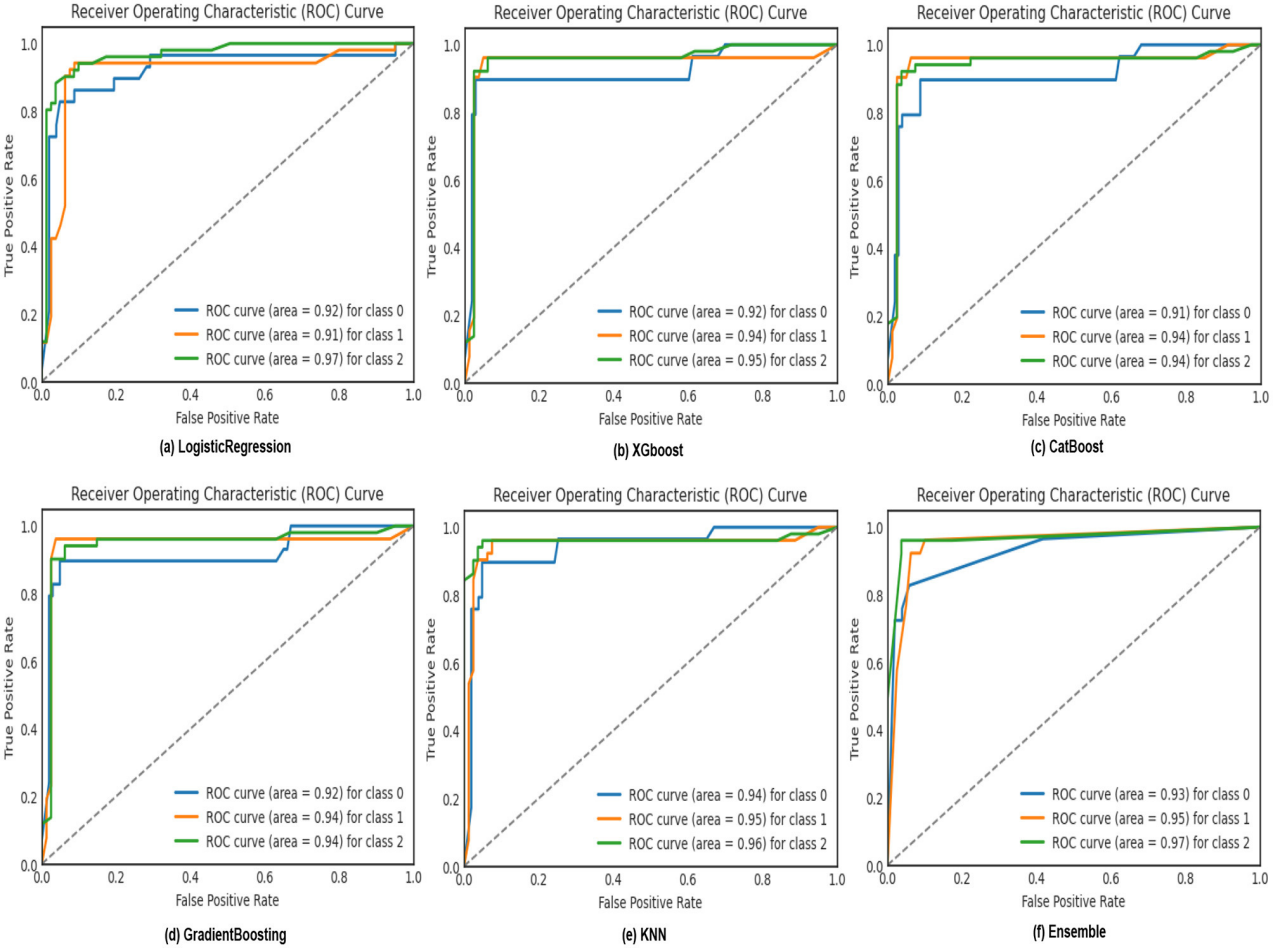
ROC curve depicting the true positive rates of logistic regression, XGBoost, CatBoost, KNN, and Gradient Boosting models for sleep disorder classification.

**Figure 9:**
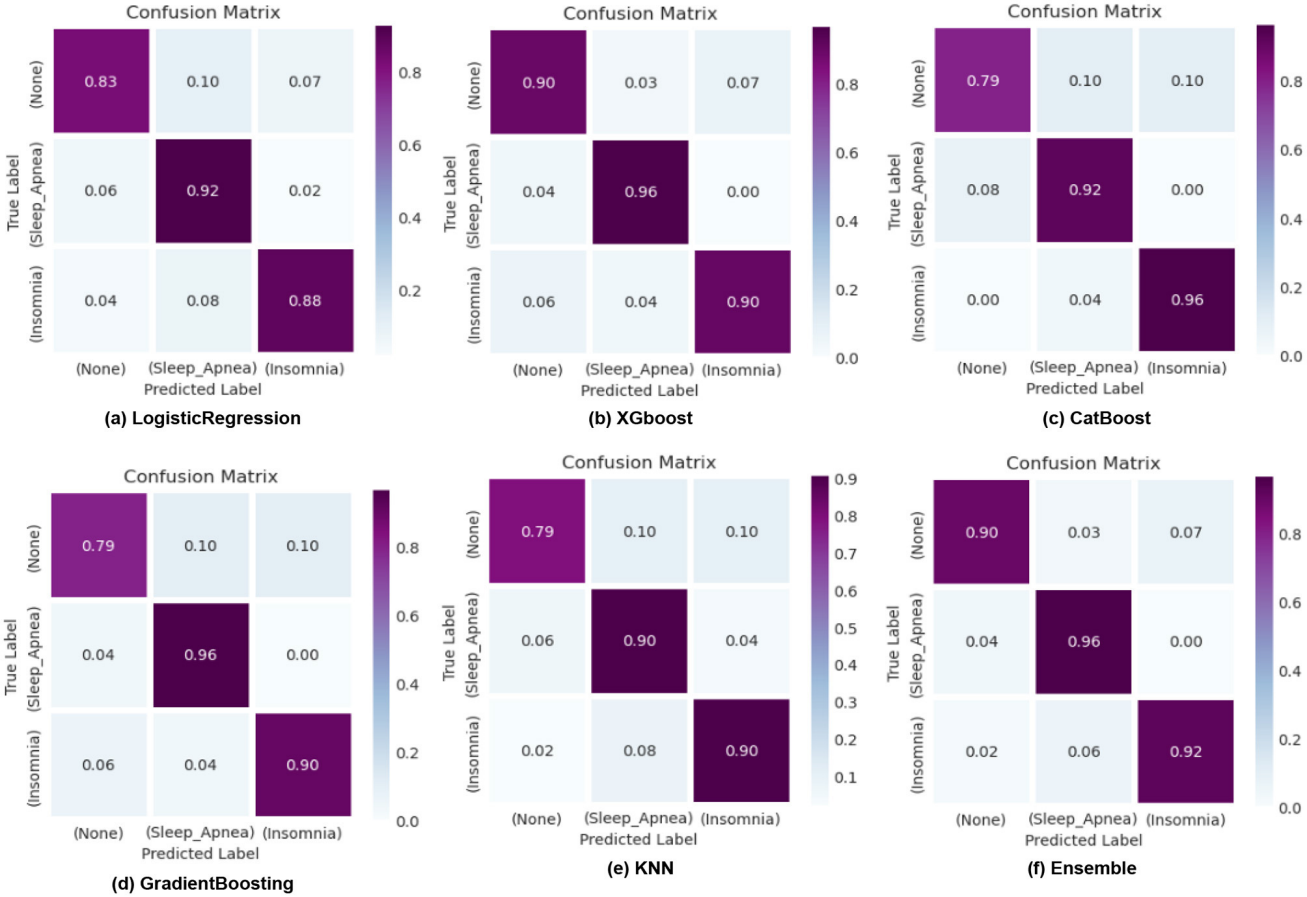
Confusion matrix illustrating the classification results of logistic regression, XGBoost, CatBoost, KNN, and Gradient Boosting models for sleep disorder detection.

ROC curves, as shown in Figure 8, are utilized in binary classification scenarios to assess and compare the performance of classification models. They are precious in situations where the balance between sensitivity and specificity and the impact of different classification thresholds needs to be carefully considered. The curve illustrates the accurate favorable rates of various models, including KNN, XGBoost, CatBoost, Logistic Regression, Gradient Boosting, and our Ensemble model. Notably, our Ensemble model outperforms the others across all three classes: 0 class with a rate of 0.93, 1st class with 0.95, and 2nd class with an impressive 0.97, corresponding to sleep apnea, insomnia, and none, respectively. Confusion matrices, as illustrated in Figure 9, serve as a cornerstone for evaluating the performance of machine learning models in sleep disorder detection. It provides a comprehensive breakdown of predictions, enabling a detailed assessment of the model's strengths and weaknesses in classifying different types of sleep disorders and negative cases. Quantifying errors and categorizing predictions offer valuable insights into model biases and imbalances, guiding optimization strategies. Additionally, the confusion matrix makes it easier to calculate critical performance metrics that are essential for assessing the efficacy of the model and improving its classification abilities, including accuracy, precision, recall, and F1-score. In the end, this iterative process in sleep medicine that is guided by the confusion matrix improves patient care and diagnostic accuracy.

The Table 5 outlines the performance metrics of a classification model across various classes after applying SMOTE, including None, Apnea, and Insomnia. These metrics, comprising Precision, Recall, and F1-score, indicate the model's effectiveness in correctly identifying instances belonging to each class. Notably, the class Apnea showcases exceptional precision, boasting a value of 0.99, which signifies a high accuracy in the model's predictions for this particular class. Additionally, the None and Apnea classes exhibit a recall rate of 0.98, indicating the model's ability to identify a significant portion of actual instances within these classes. Despite variations in precision and recall across classes, all classes maintain a consistent F1-score of 0.97, suggesting a uniform balance between precision and recall. Overall, while the model demonstrates strong performance across all classes, the notably high precision in the Apnea class underscores the model's efficacy in accurately predicting instances of this condition.

**Table 5:** Performance metrics after sprint.

**Class**	**Precision**	**Recall**	**F1-score**
None	0.97	0.98	0.97
Apnea	0.99	0.97	0.97
Insomnia	0.97	0.97	0.97

The Table 6 provides an overview of performance metrics for a classification model before applying SMOTE across different classes, namely None, Apnea, and Insomnia. These metrics include Precision, Recall, and F1-score, which are fundamental in accurately evaluating the model's ability to classify instances within each class. Notably, the Apnea class exhibits the highest precision value of 0.99, indicating a high level of accuracy in the model's predictions for this particular class. Additionally, the None class demonstrates a recall rate of 0.95. In contrast, the Apnea class achieves a recall rate of 0.96, suggesting the model can effectively identify a substantial portion of actual instances within these classes. Moreover, all classes maintain consistent F1 scores, with values ranging from 0.96 to 0.97, highlighting a balanced performance in precision and recall. The Table 7 presents the performances of various models across ten different folds. The models compared include Catboost, GradientBoost, SVM, XGBoost, Logistic Regression, KNN, and the Proposed Model. Each row represents a fold number, and each column represents a specific model. The metrics provided include precision scores for each model on each fold. Notably, the Proposed Model consistently achieves high precision scores across all folds, ranging from 0.895 to 0.995. Additionally, XGBoost and the Proposed Model demonstrate competitive performance, consistently achieving precision scores above 0.9 across most folds. Conversely, logistic regression consistently exhibits lower precision scores than other models. Overall, the table provides a comprehensive comparison of model performance, allowing for insights into the effectiveness of different algorithms across various folds.

**Table 6:** Performance measures prior to SMOTE.

**Class**	**Precision**	**Recall**	**F1-score**
None	0.97	0.95	0.96
Apnea	0.99	0.96	0.97
Insomnia	0.96	0.97	0.96

**Table 7:** Comparing the 10-fold cross-validation results of the proposed and implemented models.

**Fold**	**Catboost**	**GradientBoost**	**SVM**	**XGBoost**	**Logistic regression**	**KNN**	**Proposed model**
1	0.897	0.895	0.865	0.895	0.515	0.802	0.895
2	0.775	0.775	0.776	0.775	0.505	0.735	0.775
3	0.865	0.865	0.835	0.8655	0.685	0.796	0.865
4	0.823	0.823	0.823	0.823	0.643	0.779	0.823
5	0.965	0.965	0.923	0.965	0.707	0.965	0.965
6	0.929	0.925	0.932	0.929	0.694	0.826	0.932
7	0.895	0.895	0.867	0.863	0.505	0.855	0.893
8	0.866	0.863	0.967	0.865	0.784	0.800	0.995
9	0.925	0.942	0.931	0.889	0.686	0.932	0.953
10	0.929	0.925	0.835	0.929	0.721	0.835	0.969

### 4.2 Comparitive analysis of various work

Table 8 provides a comparative overview of methodologies employed in sleep disorder detection, detailing the techniques, signal types, and corresponding accuracies. Studies by Yadav et al. (2023) and Jarchi et al. (2020) utilized Decision Trees and various methods like SVM and Random Forest, respectively, with accuracies ranging from 72% to 93%. Zhang et al. (2021) achieved an accuracy of 96.1% with a Deep CNN-LSTM model, while Lee and Kim (2018) and Peng and Kou (2023) obtained accuracies of 69.25% and 86.63% using GRU networks and AlexNet, respectively. Notably, our proposed Ensemble model outperforms others with an accuracy of 96.88%, underscoring its effectiveness in detecting sleep disorders. This highlights the diversity of approaches in the field, with Ensemble modeling emerging as a promising method for improved accuracy.

**Table 8:** Comparative evaluation of the proposed and existing works.

**References**	**Focused methods**	**Used data**	**Accuracy**
Yadav et al. (2023)	Decision tree	ECG and EMG	93%
Jarchi et al. (2020)	LSVM, ras	ECG and EMG	72%
Zhang et al. (2021)	Deep CNN-LSTM	ECG signals	96.1%
Lee and Kim (2018)	GRU network	EOG signals	69.25%
Peng and Kou (2023)	AlexNet	ECG signals	86.63%
**Our proposed model**	Our ensemble model	Health & Lifestyle	96.88%

### 4.3 Explainable artificial intelligence (XAI)

A widely used technique is SHAP (Shapley Additive Explanations) used to understand machine learning model output. By indicating how each feature contributed to the final prediction, it aids in the interpretation of model results.

**Figure 10:**
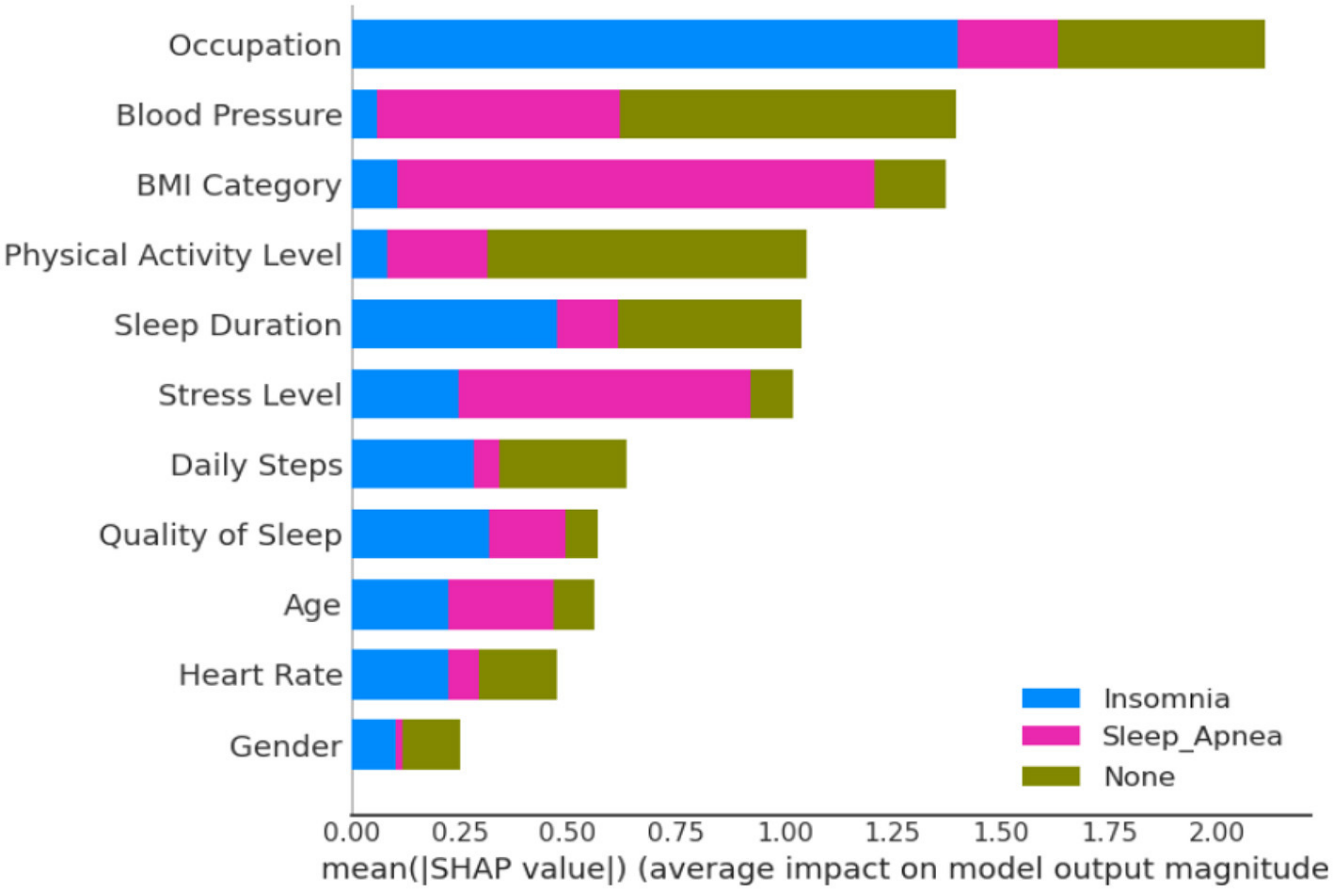
Showcasing dependencies associated with sleep-disorder related conditions, with additional insights into Explainable Artificial Intelligence (XAI).

The SHAP bar plot as depicted in Figure 10, derived from a comprehensive analysis of 11 features, including occupation, BMI, blood pressure, sleep duration, stress level, daily steps, heart rate, gender, age, and physical activity level, offers valuable insights into the dependencies associated with sleep-related conditions—insomnia, sleep apnea, or none. Notably, occupation emerges as a significant contributor to insomnia, suggesting a strong association between specific work-related factors and the likelihood of experiencing insomnia. Concurrently, BMI stands out as a critical factor linked to sleep apnea, indicating that individuals with higher BMI levels may be more susceptible to this sleep disorder. The significance of other features underscores the complexity of the relationships influencing sleep conditions. The SHAP analysis provides a nuanced understanding of the impact of each feature, offering a model-agnostic perspective on the importance of these variables in predicting various sleep-related outcomes.

## 5 Discussion

The presented ensemble model for sleep Disorder detection represents a significant advancement in predictive modeling, leveraging a strategic combination of diverse machine learning techniques. Ensemble, which includes a voting classifier, k-nearest neighbors (kNN), random forest, support vector machine (SVM), logistic regression, and XGBoost, each provides a distinct approach to improving overall predictive performance. By successfully integrating various decision-making processes, the voting and stacking strategies lessen overfitting, bias, and variance—all prevalent in standalone models. Its ability to handle noisy physiological information and adapt to complex, non-linear sleep apnea patterns accounts for its higher performance, guaranteeing more reliable and accurate predictions.

The ensemble's mechanism involves a meticulous integration of decision-making processes from each model, facilitated by voting and stacking techniques. The ensemble excels in discerning intricate patterns indicative of sleep-related conditions, achieving a remarkable accuracy rate of 96.88%. This high accuracy underscores the effectiveness of combining diverse models to overcome the challenges posed by sleep disorder detection. The interpretability of results is enhanced through softmax labels, providing valuable insights into the features contributing to predictions. Our efforts to diagnose sleep disorders are driven by the urgent need to address the significant adverse effects of sleep disorders on human health and well-being. Chronic leg movement disorder, sleep apnea, insomnia, and other sleep disorders can lead to many health problems, such as heart problems, memory loss, and reduced quality of life. Polysomnography tests are expensive and time-consuming in traditional diagnostic methods, which limits access to diagnosis and treatment. Our exploration into detection tools for sleep problems stems from their profound impact on individuals' health and overall well-being. Sleep difficulties can result in cognitive impairment, hindered performance in daily tasks, emotional fluctuations, and compromised stress management. Moreover, they are often associated with an elevated risk of mental health issues like depression and physical illnesses such as diabetes and obesity. We aim to mitigate the detrimental effects of sleep problems by implementing efficient detection systems. The ensemble model's scalability and computing viability are essential for practical uses. Although integrating several algorithms in ensemble models always increases computational costs, these difficulties can be lessened by using hardware accelerators like GPUs and TPUs and strategies like model pruning and compression. These improvements guarantee that the model can satisfy the requirements for practical use. The ensemble model's resilience across various datasets or unseen data is crucial. Sophisticated data augmentation and cross-validation techniques are incorporated to guarantee generalizability during training. The model's capacity to adjust to unknown situations is further reinforced by using domain adaptation techniques and broadening the dataset to encompass a variety of physiological and demographic characteristics. This guarantees steady functioning under a range of real-world circumstances.

However, like any model, the ensemble has its limitations. One notable limitation is the dependence on the quality and diversity of the training data. If sufficiently representative, the model may generalize to unseen cases. Additionally, the interpretability of ensemble models, while improved compared to individual models, may still need to be improved in fully understanding the intricate relationships between features. For future directions, refining the ensemble model by incorporating more advanced deep-learning architectures could further enhance its ability to capture complex patterns. Moreover, expanding the dataset to include a more diverse range of demographic and physiological factors could contribute to a more comprehensive understanding of sleep-related conditions. Regarding model details, the ensemble mechanism combines individual models' outputs through a voting classifier. Each model's decision-making process is weighted based on its contribution to the overall accuracy. The accuracy of 96.88% is achieved through the collaborative strength of the ensemble, showcasing its robustness in identifying sleep disorder events.

## 6 Conclusions

The presented ensemble model for sleep disorder detection, utilizing machine learning techniques such as Random Forest, SVM, logistic regression, KNN, XGBoost, and a voting classifier, demonstrates a high accuracy of 96.88%. The model's interpretability, achieved through softmax labels and a thorough analysis of 11 features, enhances understanding of sleep-related conditions. While celebrating its success, it is crucial to acknowledge limitations related to training data quality and ensemble decision interpretation. The model contributes significantly to healthcare by providing a powerful diagnostic tool, and future work should focus on refining and expanding its capabilities. Through our proposed methodology, we want to attenuate the negative impacts of sleep disorders, improve overall health outcomes, and promote well-being by managing them through effective identification. The precision achieves across various classes, including insomnia, sleep apnea, and none, underscores its versatility. Despite its success, ongoing efforts are necessary to address real-world deployment and data generalization challenges. This ensemble model is a promising step forward in machine learning and healthcare, offering a reliable tool for accurately diagnosing sleep disorders. In conclusion, the ensemble model achieves superior accuracy in sleep disorder detection and provides valuable insights into the underlying patterns. Its strategic combination of models, interpretability, and high accuracy make it a promising tool for healthcare professionals. As we look to the future, addressing limitations and incorporating advancements in deep learning will further solidify the ensemble's position as a leading solution in sleep disorder diagnosis.

## Data Availability

The datasets presented in this study can be found in online repositories. The names of the repository/repositories and accession number(s) can be found at: https://www.kaggle.com/datasets/uom190346a/sleep-health-and-lifestyle-dataset.
